# Supplementary material for: Impact of dhps mutations on sulfadoxine-pyrimethamine protective efficacy and implications for malaria chemoprevention
Source: Nat Commun. 2025 May 8;16:4268. doi: 10.1038/s41467-025-58326-z (PMC12062426; doi:10.1038/s41467-025-58326-z)
Supplement: Supplementary file 1 — Supplementary Information [file 41467_2025_58326_MOESM1_ESM.pdf]

Supplementary Information

Impact of *dhps* mutations on sulfadoxine-pyrimethamine protective efficacy and implications for malaria chemoprevention

Contents

Supplementary Table 1: Inclusion criteria and PCR methods in included studies 2
Supplementary Table 2: Availability of data on *dhps* markers. 4
Supplementary Note 1: Model structure 5
Supplementary Figure 1: Estimated genotype frequencies 7
Supplementary Figure 2: Predicted mean duration of protection against each genotype by drug arm... 8
Supplementary Table 3: Prior distributions and posterior estimates... 9
Supplementary Table 4: Predicted 30-day protective efficacy for each trial arm 11
Supplementary Table 5: Validation analyses of IPTi trial data 12
Supplementary Note 2: Exploring the effects of drug concentrations and initial parasite density 13
Supplementary Figure 3: Protective efficacy and duration of protection for SPAQ, CQ and SP-CQ .. 15
Supplementary Note 3: Web interface 16
Supplementary Figure 4: Frequency estimates of dhps haplotypes across sub-Saharan Africa 17
Supplementary Figure 5: Impact of a single SP dose across sub-Saharan Africa 18
Supplementary Table 6: Model diagnostics 19
Supplementary Table 7 Sensitivity analysis accounting for heterogeneity in risk of transmission. 21
Supplementary Figure 6: Estimated incidence of malaria infection 22
References 23

Supplementary Table 1: Inclusion criteria and PCR methods in included studies

Publication	Site, Country, Year	Inclusion criteria	PCR methods / assumptions
Bell et al., 2008	Blantyre, Malawi, 2003-2005	Inclusion criteria were: i) age ≥12 and <60 months, ii) weight ≥6 kg, iii) axillary temperature ≥37.5°C, iv) no history of treatment with an antimalarial, cotrimoxazole or a tetracycline antibiotic in the previous week, v) no features suggesting severe malaria or a concomitant illness, vi) haemoglobin ≥5.0 g/dl using Hemocue®, and vii) P. falciparum monoinfection with a parasite density between 2000 and 200,000 parasites per µl.	DNA was extracted and a nested PCR was used to identify msp2 polymorphisms. Parasites were classified as recrudescent if they shared any of the bands that were present on day 0 and as reinfections if they had no bands in common. Nested PCR followed by mutation-specific restriction enzyme digestion was used to determine the prevalence of different alleles in the *dhfr*, *dhps*, *pfcrt* and *pfmdr1* genes in day 0 parasites and parasites appearing at any time from day 12 onwards after treatment.
Gesase et al., 2009	Tanga Region, Northern Tanzania, 2006	Children aged 6-59 months with history of fever,a positive blood smear (>2,000 and 200,000 asexual parasites per microliter), had no signs of severe malaria or malnutrition, and were not enrolled in the IPTi trial or had recently received an antimalarial. A separate cohort of asymptomatic children aged 2-10 months, which was part of the original publication was not included in our analysis.	Multiplicity of infection (MoI) was assessed by examining the numbers of alleles detected at MSP2 and pfPK2. Where the number of alleles at these two loci differed, the higher of the two values was used since this is the minimum number co-infecting genotypes which can explain the observed diversity. For the purposes of our study, we reclassified reinfection based on whether the MOI was higher on the day of failure compared to day 0. There were 17 “reinfections”, which occurred between day 1 and day 3, which we assumed to be either latent or missed infections on day 0. These were excluded and censored in the previous observed time point. On the day of failure only the following *dhps* haplotypes were observed: SGEAA, SGEGA and mixed (SGEAA+SGEGA). Despite a few *dhps* wildtype genotypes being observed on day 0, they all cleared following SP treatment and thus none were observed in new infections. If the *dhps* haplotype on both day 0 and day of failure was mixed (SGEAA+SGEGA) and there was both a recrudescence and reinfection on day of failure, then this was classified as “undetermined infection”, as a haplotype could not be assigned to a new infection (N=7).
Nahum et al., 2007; Nahum et al., 2009	Cotonou, Benin,2003-2005	Children aged 6-59 months with fever (axillary temperature ≥37.5°C), a P. falciparum mono-infection, with a parasite density between 1,000–200,000/µL, a PCV≥15% and without symptoms of severe or other concomitant illness	DNA was purified and genotyping done by nested PCR for variable blocks within the merozoite surface protein 1 and 2 (*msp1* and *msp2*). A recrudescence was defined when at least one common band was observed for both markers in the day 0 sample and at the day of recurrent parasitaemia.
Allen et al., 2009 EKDTF	Magude, Mozambique, 2004-2005	Individuals of any age older than one year with fever and parasitaemia up to 500,000 asexual parasites/µl blood were recruited. Those with recent (one week) history of	Dried blood samples were collected and *P.falciparum* DNA was extracted and based on variations in *msp1*, *msp2* and *Glurp*, treatment failures were classified as a re-infection or a recrudescence. Infections were classified as recrudescent if PCR products for all three markers from day 0 and day

	Boane, Mozambique, 2004-2005<br>Namaacha, Mozambique, 2003<br>Catuane, Mozambique, 2003	antimalarials or folates were excluded, as were patients with severe malaria symptoms or other severe illness.	of failure parasites were identical. If the banding patterns for any markers differed between day 0 and day of failure parasites, the infection was classified as a re-infection. Although the last day of active follow-up was day 42, one reinfection in the SP group was observed past day 42 though passive case detection. We censored observations on day 42 to ensure consistency in the probability of detection. Additionally, reinfections were censored if they occurred within the first 3 days following treatment (1 in SP group and 1 in SPAS group), as these are likely to be due to recrudescence.
Barnes et al., 2006	Namaacha, Mozambique, 2002, RHXNJ<br>Bela Vista, Mozambique, 2002, RZENT<br>Bela Vista, Mozambique, 2003, NMMSB	Individuals of any age older than one year with fever and parasitaemia up to 500,000 asexual parasites/µl blood were recruited. Those with recent (one week) history of antimalarials or folates were excluded, as were patients with severe malaria symptoms or other severe illness.	Dried blood samples were collected and P.falciparum DNA was extracted and based on variations in *msp1*, *msp2* and *Glurp*, treatment failures were classified as a re-infection or a recrudescence. Infections were classified as recrudescent if PCR products for all three markers from day 0 and day of failure parasites were identical. If the banding patterns for any markers differed between day 0 and day of failure parasites, the infection was classified as a re-infection.
Barnes et al., 2008,Mabuza et al., 2005	Mpumalanga, South Africa, 2002,PEADD	Inclusion criteria: Age 2 years or above, symptomatic uncomplicated P. falciparum infection with an asexual parasite density above 1 000 parasites/µl blood, proximity of patient's home for follow-up, informed consent, and history of fever or axillary temperature above 37.5°C.Exclusion criteria included severe malaria, intolerance of oral therapy, and pregnancy	Polymerase chain reaction (PCR) amplification of the polymorphic genetic markers *msp1*, *msp2* and *glurp1* was used to differentiate between true recrudescence and new infections.
Bredenkamp et al., 2001	Ndumu, KwaZulu-Natal, South Africa, 2000	Age 2 or over with clinical malaria (1996 WHO criteria)	Polymerase chain reaction (PCR) amplification of the polymorphic genetic markers *msp1*, *msp2* and *glurp1* was used to differentiate between true recrudescence and new infections.

Refer to individual publications for more details on the criteria/methodology adopted by each study.

Supplementary Table 2: Availability of data on *dhps* markers.

Publication	Site, Country, Year	*Day 0* Number at risk	*Day 0* full *dhps* haplotype	*Day 0* % incomplete	*Day of reinfection* Number reinfected	*Day of reinfection* full *dhps* haplotype	*Day of reinfection* % incomplete	Assumptions on frequency of *dhps* markers (A437G-K540E-A581G)
Bell et al., 2008	Blantyre, Malawi, 2003-2005	455	47	89.7%	87	0	100.0%	Assumed to be *dhps* GEA fixed based on 97% prevalence on day 0
Gesase et al., 2009	Tanga Region, Northern Tanzania, 2006	87	58	33.3%	32	25	21.9%	
Nahum et al., 2007; Nahum et al., 2009	Cotonou, Benin,2003-2005	237	0	100.0%	14	0	100.0%	Assumed to be *dhps* GKA (437G-K540-A581) fixed based on ~85% prevalence on day 0 (individual-level data not available)
Allen et al., 2009 EKDTF	Magude, Mozambique, 2004-2005	179	158	11.7%	20	0	100.0%	
	Boane, Mozambique, 2004-2005	104	92	11.5%	5	0	100.0%	
	Namaacha, Mozambique, 2003	78	63	19.2%	1	0	100.0%	
	Catuane, Mozambique, 2003	47	41	12.8%	0	0	NA	
Barnes et al., 2006	Namaacha, Mozambique, 2002, RHXNJ	97	70	27.8%	8	3	62.5%	
	Bela Vista, Mozambique, 2002, RZENT	49	44	10.2%	3	0	100.0%	
	Bela Vista, Mozambique, 2003, NMMSB	25	24	4.0%	1	1	0.0%	
Barnes et al., 2008,Mabuza et al., 2005	Mpumalanga, South Africa, 2002,PEADD	152	58	61.8%	0	0	NA	
Bredenkamp et al., 2001	Ndumu, KwaZulu-Natal, South Africa, 2000	129	0	100.0%	4	0	100.0%	Assumed to be *dhps* GEA fixed based on ~90% prevalence of 540E in an unpublished survey in the same year and site (Ndumu, 1999)
	Total	**1639**	**655**	**60.0%**	**175**	**29**	**83.4%**	

Supplementary Note 1: Model structure

A model simulating the infection dynamics in each trial was fitted to the trial data using Bayesian MCMC methods detailed in a previous publication.[1] We model the incidence of infection over time (t) since receiving chemoprevention as:

$$\Lambda_C(\mathrm{t}) = \Lambda\left(1 - e^{-\left(\frac{t}{\lambda}\right)^{w}}\right)$$

where Λ is the force of infection and $e^{-\left(\frac{t}{\lambda}\right)^{w}}$ is the probability of protection provided by the drug at each time point since receiving the SP dose. This probability is assumed to follow a Weibull survival curve with a scale parameter λ and a shape parameter w. A separate force of infection was estimated for each site.

The above model can be extended to account for multiple genotypes present in a single site, and different probability of protection against each genotype. The probability that an individual becomes infected with each strain X at each time step is estimated as:

$$\Lambda_{C_X}(\mathrm{t}) = \Lambda F_X\left(1 - e^{-\left(\frac{t}{\lambda_X}\right)^{w_X}}\right)$$

where

$X = triple, quadruple, quintuple, sextuple$

and F_X is the frequency of the strain in the parasite population.

The mean duration of protection from a new infection with a given strain (X) can also be determined from the Weibull curve as:

$$Mean \text{ duration of protection} = \lambda_X\, \Gamma\, \left(1 + \frac{1}{w_X}\right)$$

The model assumes that only one type of parasite strain can be acquired during each time step, and accounts for the fact that some infections will not be genotyped at a particular codon position due to failure to diagnose by polymerase chain reaction methods. The full model methods and deterministic equations describing the proportion of individuals in each state (uninfected and infected with each genotype) are detailed in a previous publication[1]. The number of those who remain uninfected and new infections with each parasite strain at each time point are assumed to be multinomially distributed with probabilities $\frac{U(t)}{U(t-dt)}$ and $\frac{I_X(t)-I_X(t-dt)}{U(t-dt)}$, where t is continuous time, dt is the time step, U is the proportion uninfected , and I_X is the cumulative proportion infected with a particular genotype X.

In areas where we expect a given genotype to be fixed, the overall protection estimated using the equation above may represent the protection against that genotype. In the study conducted in Malawi [2], the frequency of the *dhps* GEA mutant estimated at day 0 (before receiving a drug) was 97% when analysing infections which were unmixed at the relevant *dhps* loci. For the study conducted in KwaZulu-Natal, South Africa, the genotype of the reinfections was not available. However unpublished data from the same area (Ndumo) and year as the trial, suggested that the prevalence of the *dhps* 540E mutation was >90% (data contained within [3]). For these reasons we used a one-strain model for these two studies with the overall duration of protection corresponding to the protection against the quintuple genotype by fitting a single Weibull survival curve.

Similarly, we used a one-strain model for the study conducted in Benin, where the frequency of the *dhps* GKA (*437G-540K-581A*) mutant prior to treatment was 85% but individual-level data for this study were not available. The overall duration of protection for this study corresponds to that against the *dhps* GKA genotype.

A two-strain model was used for the study carried out in Northern Tanzania [4], where complete haplotype information was available, and all new infections consisted of either quintuple or sextuple genotypes. We also used a two-strain model for the studies carried out in Mozambique and the one study carried out in South Africa [5-8] (mixture of A437-K540-A581 and 437G-540E-A581). In the Mozambique and South Africa studies the *dhps* GKA mutant was either completely absent or very rare (≤5% in all studies and <1% in most studies). If the genotype was undetermined, this was included in the model, and those infections still contributed to the

estimation of malaria incidence. The modelling of "undetermined" new infections is explained in full elsewhere.[1]

Due to incomplete data on the *dhps* genotype on the day of reinfection, the frequency of *dhps* genotypes on day 0 (prior to treatment) was used for estimation of F_X. We assumed that the number of samples with each genotype (n_X) on day 0 was binomially distributed with probability F_X.

$n_X \sim binomial(N_X, F_X)$,

Where N_X is the total number of samples with unmixed infections in the relevant *dhps* loci (437, 540 and 581).

All parameters were estimated simultaneously in a single combined model with:

1) site-specific force of infection,
2) site-specific *dhps* genotype frequencies and
3) genotype- and drug-specific protection parameters (λ and w).

The estimated parameters from the combined fit are shown in the table below (Supplementary Table 3)

Supplementary Figure 1: Estimated genotype frequencies

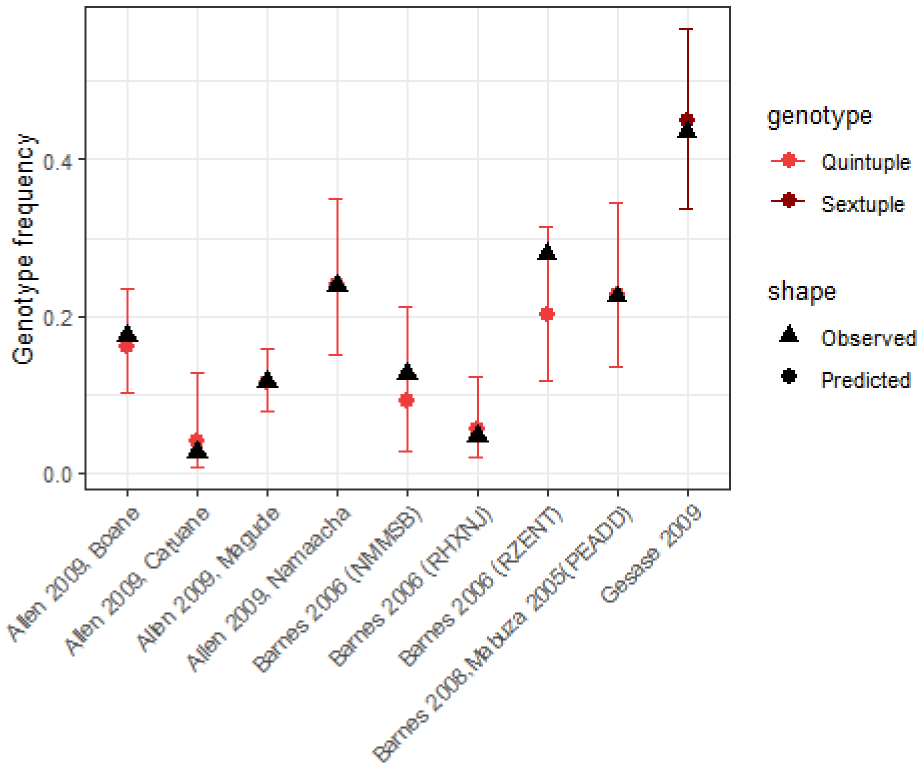

Black triangles denote the observed genotype frequency on day 0. The predicted median posterior frequencies and 95% credible intervals are shown in red for the quintuple and in dark red for the sextuple (dhps GEG (437G-540E-581G). Sample sizes for study sites with available *dhps* haplotype data on day 0 from left to right: 92, 41, 158, 63, 24, 70, 44, 58, 58.

Supplementary Figure 2: Predicted mean duration of protection against each genotype by drug arm.

Estimates are based on individual participant data with a total sample size of 1,639 across 12 trial sites. Points denote posterior medians and error bars represent 95% credible intervals). Different colours denote different genotypes. The *dhps* AKA genotype indicates the sulfadoxine susceptible genotype with no *dhps* mutations. Gene names are shown in italics and mutations are underlined: *dhps* AKA (A437-K540-A581), *dhps* GKA (437G-K540-A581) in, *dhps* GEA (437G-540E-A581), *dhps* GEG (437G-540E-581G); *dhps: dihydropteroate synthase;* SP: sulfadoxine pyrimethamine, SPAS: SP + artesunate, SPAQ: SP + amodiaquine, CQ: chloroquine

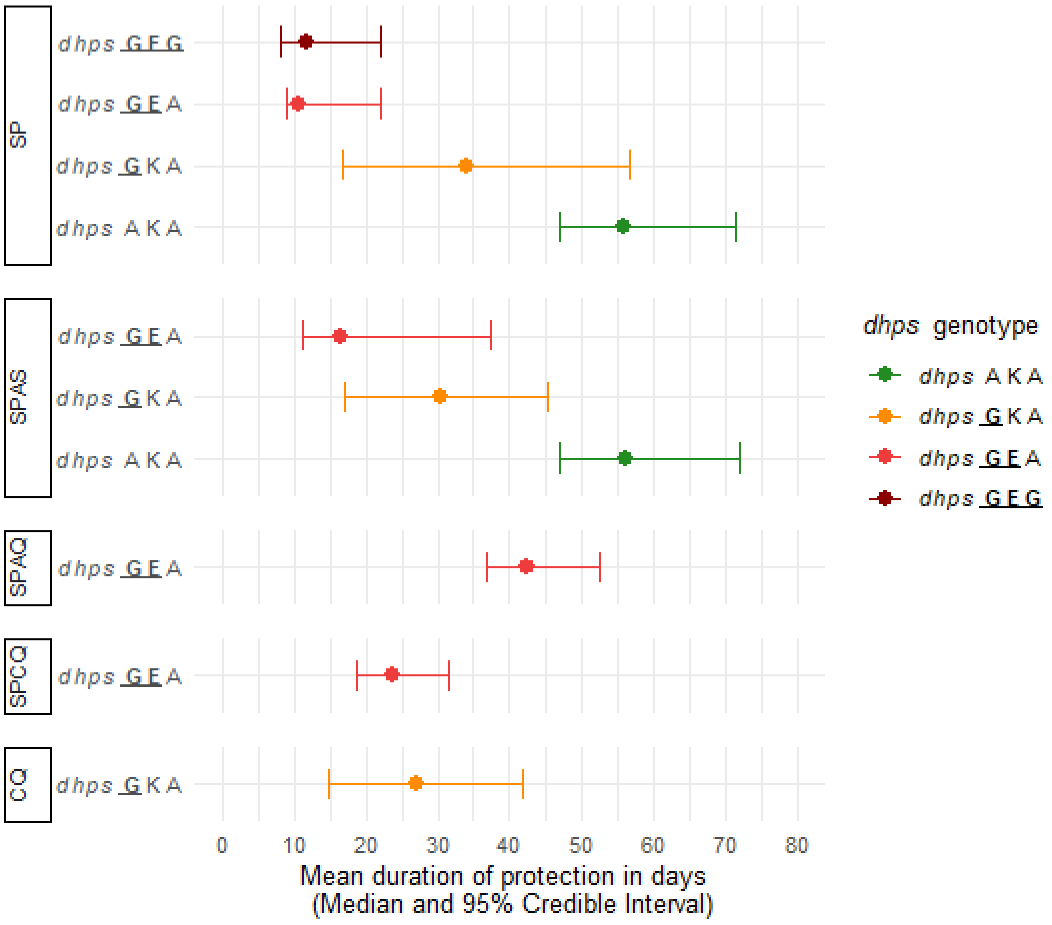

Supplementary Table 3: Prior distributions and posterior estimates

Parameter	*Dhps* genotype (for genotype-specific parameter)	Drug (for drug-specific parameters)	Site (for site-specific parameters)	Prior distribution *	Posterior estimate and 95% Credible Interval	Units	Source (for prior)
Malaria Incidence	−	−	Blantyre, Malawi, 2003-2005 (Bell et al., 2008)	gamma(4.8,1.0) mean=4.9, σ^2=5	6.53 (4.84,9.49)	Malaria Infections per person per year	[9-11]
	−	−	Tanga Region, Northern Tanzania, 2006 (Gesase et al., 2009)	gamma(30,0.4) mean=30, σ^2=10	27.46(20.45,36.54)		[4,9-11]
	−	−	Cotonou, Benin, 2003-2005 (Nahum et al., 2007; Nahum et al., 2009)	gamma(3.2,1.2) mean=4.0, σ^2=5	0.68(0.39,1.10)		[9-11]
	−	−	Magude, Mozambique, 2004-2005 (Allen et al., 2009)	gamma(60.6,0.3) mean=17.4, σ^2=5	16.67(12.96,21.01)		[5,9,10]
	−	−	Boane, Mozambique, 2004-2005 (Allen et al., 2009)	gamma(17.7,0.5) mean=9.4, σ^2=5	7.80(4.88,11.82)		[5,9,10]
	−	−	Namaacha, Mozambique, 2003 (Allen et al., 2009)	gamma(0.7,2.6) mean=1.9, σ^2=5	0.93(0.11,3.52)		[5,9,10]
	−	−	Catuane, Mozambique, 2003 (Allen et al., 2009)	gamma(0.1,7.1) mean=0.7, σ^2=5	0.002(0.000,3.17)		[5,9,10]
	−	−	Namaacha, Mozambique, 2002 (RHXNJ) (Barnes et al., 2006)	gamma(0.7,2.6) mean=1.9, σ^2=5	12.77(4.00,39.35)		[5,9,10]
	−	−	Bela Vista Mozambique, 2002 (RZENT) (Barnes et al., 2006)	gamma(44.4,0.3) mean=14.9, σ^2=5	13.09(9.50,17.46)		[9-11]
	−	−	Bela Vista, Mozambique, 2003 (NMMSB) (Barnes et al., 2006)	gamma(25.1,0.4) mean=6.7, σ^2=5	10.24(6.75,14.79)		[9-11]
	−	−	Mpumalanga, South Africa, 2002 (PEADD) (Barnes et al., 2008 ; Mabuza et al., 2005)	gamma(0.1,7.1) mean=0.7, σ^2=5	0.0003(0.0000,0.36)		[9-11]
	−	−	Ndumu, KwaZulu-Natal, South Africa, 2000 (Bredenkamp et al., 2001)	gamma(0.1,7.1) mean=0.7, σ^2=5	2.31(0.69,5.61)		[9,10,12]

* To inform the prior distribution we used the malariaEquilibrium package in R (https://github.com/mrc-ide/malariaEquilibrium) and assumed 45% treatment coverage. We also assumed that 60% of EIR results to a successful infection. The incidence or prevalence reported in the original publications was used where possible and where unavailable, MAP prevalence was used to calibrate a malaria transmission model developed by Imperial College London.

Supplementary Table 3 (continued): Prior distributions and posterior estimates

Parameter	Dhps genotype (for genotype-specific parameter) A437G-K540E-A581G	Drug (for drug-specific parameters)	Site (for site-specific parameters)	Prior distribution	Posterior estimate and 95% Credible Interval	Units	Source (for prior)
Frequency	GEA	−	Magude, Mozambique, 2004-2005	uniform(0,1)	0.11(0.08,0.16)	Proportion	−
	GEA	−	Boane, Mozambique, 2004-2005	uniform(0,1)	0.16(0.1,0.23)		−
	GEA	−	Namaacha, Mozambique, 2003	uniform(0,1)	0.24(0.15,0.35)		−
	GEA	−	Catuane, Mozambique, 2003	uniform(0,1)	0.04(0.01,0.13)		−
	GEA	−	Namaacha, Mozambique, 2002 (RHXNJ)	uniform(0,1)	0.06(0.02,0.12)		−
	GEA	−	Bela Vista Mozambique, 2002 (RZENT)	uniform(0,1)	0.2(0.12,0.31)		−
	GEA	−	Bela Vista, Mozambique, 2003 (NMMSB)	uniform(0,1)	0.09(0.03,0.21)		−
	GEA	−	Mpumalanga, South Africa, 2002 (PEADD)	uniform(0,1)	0.23(0.14,0.35)		−
	GEG	−	Tanga Region, Northern Tanzania, 2006	uniform(0,1)	0.45(0.34,0.57)		−
Probability of determining genotype for new infection	−	−	Tanga Region, Northern Tanzania, 2006	uniform(0,1)	0.77(0.61,0.89)	Probability	−
Scale parameter (λ) corresponding to the Weibull distribution of protective efficacy over time	AKA	SP	−	gamma(7,4)	59.07(47.88,81.12)	−	−
	GKA		−	gamma(7,4)	36.91(15.93,66.43)	−	−
	GEA		−	gamma(7,4)	11.92(7.52,18.27)	−	−
	GEG		−	gamma(7,4)	12.86(8.03,27.06)	−	−
Shape parameter (w) corresponding to the Weibull distribution of protective efficacy over time	AKA		−	gamma(4,1.25)	8.38(4.49,15.07)	−	−
	GKA		−	gamma(4,1.25)	4.89(1.56,11.34)	−	−
	GEA		−	gamma(4,1.25)	1.92(1.07,3.68)	−	−
	GEG		−	gamma(4,1.25)	3.61(1.1,10.04)	−	−
Scale parameter (λ)	AKA	SPAS	−	gamma(7,4)	59.41(47.67,81.7)	−	−
	GKA		−	gamma(7,4)	32.96(16.41,52.73)	−	−
	GEA		−	gamma(7,4)	18.43(11.52,42.4)	−	−
Shape parameter (w)	AKA		−	gamma(4,1.25)	8.44(4.59,15.18)	−	−
	GKA		−	gamma(4,1.25)	4.91(1.7,11.06)	−	−
	GEA		−	gamma(4,1.25)	2.56(0.9,8.86)	−	−
Scale parameter (λ)	GKA	CQ	−	gamma(7,4)	29.39(13.92,49.46)	−	−
Shape parameter (w)	GKA		−	gamma(4,1.25)	4.71(1.51,11.08)	−	−
Scale parameter (λ)	GEA	SPCQ	−	gamma(7,4)	25.89(19.31,37.29)	−	−
Shape parameter (w)	GEA		−	gamma(4,1.25)	4.63(2.07,10.86)	−	−
Scale parameter (λ)	GEA	SPAQ	−	gamma(7,4)	45.65(38.08,60.59)	−	−
Shape parameter (w)	GEA		−	gamma(4,1.25)	6.26(3.16,12.14)	−	−

Supplementary Table 4: Predicted 30-day protective efficacy for each trial arm

Estimates are based on individual participant data with a total sample size of 1,639 across 12 trial sites. The estimated 30-day protective efficacy against first infection was computed for each trial arm using the posterior distribution of the estimated malaria incidence, genotype frequency and genotype-specific protection (scale and shape) parameters. 30-day protective efficacy against first infection was estimated as the percentage of new infections prevented by the drug compared to no chemoprevention. The *dhps* AKA genotype indicates the sulfadoxine susceptible genotype with no *dhps* mutations. Gene names are shown in italics and mutations are underlined: *dhps* AKA (A437-K540-A581), *dhps G*KA (437*G*-K540-A581) in, *dhps GE*A (437*G*-540*E*-A581), *dhps GEG* (437G-540E-581*G*); *dhps: dihydropteroate synthase;* SP: sulfadoxine pyrimethamine, SPAS: SP + artesunate, SPAQ: SP + amodiaquine, SPCQ: SP + chloroquine, CQ: chloroquine

**Publication**	**Site, Country, Year**	**Drug arms (N)**	**Estimated malaria incidence ppy (95% Credible Interval)**	**Estimated frequency**	**Estimated 30-day protective efficacy (95% Credible Interval)**
Bell et al., 2008	Blantyre, Malawi, 2003-2005	SP (N=114)<br>SPAS (N=114)<br>SPAQ(N=114)<br>SPCQ (N=113)	6.53 (4.84,9.49)	GEA (fixed)	SP: 28.6% (17.7%-42.2%)<br>SPAS: 46.7%(29.4%-69.5%)<br>SPAQ : 98.7%(93.8%- 99.9%)<br>SPCQ : 71.7%(53.0%-85.8%)
Gesase et al., 2009	Tanga Region, Northern Tanzania, 2006	SP(N=87)	27.46(20.45,36.54)	sextuple: 0.45(0.34,0.57), remaining=GEA	SP: 15.4% (10.0%-24.1%)
Nahum et al., 2007;<br>Nahum et al., 2009	Cotonou, Benin, 2003-2005	SP (N=77)<br>SPAS (N=81)<br>CQ (N=79)	0.68(0.39,1.10)	GKA (fixed)	SP: 92.2% (46.9% -100.0%)<br>SPAS: 88.8%(48.7% – 99.5%)<br>CQ: 82.2% (41.1%-99.1%)
Allen et al., 2009<br>EKDTF	Magude, Mozambique, 2004-2005	SP (N=93)<br>SPAS (N=86)	16.67(12.96,21.01)	GEA: 0.11(0.08,0.16), remaining=AKA	SP: 87.1% (82.2%-91.1%)<br>SPAS: 90.9% (85.9%- 95.0%)
	Boane, Mozambique, 2004-2005	SP (N=41)<br>SPAS (N=63)	7.80(4.88,11.82)	GEA: 0.16(0.1,0.23), remaining=AKA	SP: 86.4% (79.7%-91.4%)<br>SPAS: 90.5% (84.0%-95.2%)
	Namaacha, Mozambique, 2003	SP (N=40)<br>SPAS (N=38)	0.93(0.11,3.52)	GEA: 0.24(0.15,0.35), remaining=AKA	SP: 83.7% (75.2%-90.3%)<br>SPAS: 88.7% (79.8%, 94.8%)
	Catuane, Mozambique, 2003	SP (N=24)<br>SPAS (N=23)	0.002(0.000,3.17)	GEA: 0.04(0.01,0.13), remaining=AKA	SP: 97.3% (91.4%-99.8%)<br>SPAS: 98.1% (93.4%-99.8%)
Barnes et al., 2006	Namaacha, Mozambique, 2002<br>RHXNJ	SP (N=97)	12.77(4.00,39.35)	GEA: 0.06(0.02,0.12), remaining=AKA	SP: 94.0% (88.8%-97.3%)
	Bela Vista Mozambique, 2002<br>RZENT	SP(N=49)	13.09(9.50,17.46)	GEA: 0.2(0.12,0.31), remaining=AKA	SP: 80.2%(70.1%–88.1%)
	Bela Vista, Mozambique, 2003<br>NMMSB	SP(N=25)	10.24(6.75,14.79)	GEA: 0.09(0.03,0.21), remaining=AKA	SP: 91.4% (80.8%-97.3%)
Barnes et al., 2008<br>Mabuza et al., 2005	Mpumalanga, South Africa, 2002<br>PEADD	SP (N=152)	0.0003(0.0000,0.36)	0.23(0.14,0.35), remaining=AKA	SP: 85.2% (76.4%-92.2%)
Bredenkamp et al., 2001	Ndumu, KwaZulu-Natal, South Africa, 2000	SP(N=129)	2.31(0.69,5.61)	GEA (fixed)	SP: 32.2% (20.1%-47.2%)

Supplementary Table 5: Validation analyses of IPTi trial data

The sample size was 1,497 for Mozambique (SP= 747 and placebo=750) and 639 for Tanzania (SP= 319 and placebo=320). pppy: per person per year, CrI: credible interval

IPTi trial	Control group	Incidence assumption	Estimated incidence (pppy) Median (95%CrI)	Estimated mean duration of SP protection in validation data (days) Median (95%CrI)	Expected* mean duration of protection using parameter estimates from main analysis (days)
Macete et al 2006, Mozambique	**Included**	**Time varying**			
		**Week 1**	0.12(0.001-0.48)	25.0(12.0-41.5)	22.1
		**Week 2**	0.38(0.11-0.86)		
		**Week 3**	0.16(0.04-0.45)		
		**Week 4**	0.38(0.17-0.75)		
		**Week 5**	0.18(0.06-0.40)		
	**Included**	**Constant**	0.25(0.16-0.38)	24.4(11.2-42.4)	22.1
	**Excluded**	**Constant**	0.27(0.08-1.23)	20.7 (5.9-45.3)	22.1
Gosling et al 2009, Tanzania	**Included**	**Time varying**			
		**Week 1**	0.10(0.005-0.55)	10.9(3.8-29.8)	13.7
		**Week 2**	0.01(0.001-0.16)		
		**Week 3**	0.06(0.003-0.36)		
		**Week 4**	0.14(0.02-0.49)		
		**Week 5**	0.05(0.003-0.27)		
	**Included**	**Constant**	0.11(0.04-0.24)	11.1(3.5-29.2)	13.7
	**Excluded**	**Constant**	0.20(0.04-1.04)	14.9(4.0-40.7)	13.7

*****

Supplementary Note 2: Exploring the effects of drug concentrations and initial parasite density

Drug concentrations were only available in one study.[2,13] For this study, we summarise the mean and median concentrations of sulfadoxine and pyrimethamine on day 0 after receiving the drug and on day 7 by reinfection outcome (Table 1). We also summarise mean and median parasitaemia by reinfection outcome, separately for SP and SPAS treatment arms (Table 2).

We fit a Cox proportional hazards regression model to investigate the effect of drug concentration and initial parasite density on to reinfection (Table 3). We observed no significant association between the rate of new infection and initial parasitaemia or drug concentration.

Table 1- A summary of the mean and median concentrations of sulfadoxine and pyrimethamine on day 0 after receiving the drug and on day 7 by reinfection outcome (combined for SP and SPAS groups)

Day	Outcome	Drug	N	Mean concentration	Confidence Interval	Median	2.5th -97.5th percentiles
0	Reinfected	SDX	27	72.4	53.6-92.3	74.4	5.1-158.2
0	Not reinfected	SDX	71	74.2	63.1-85.4	71.5	8.3-161.6
0	Reinfected	PYM	21	290.3	229.0-351.6	253.2	95.6-501.6
0	Not reinfected	PYM	64	335.4	289.2-381.7	335.1	47.7-696.4
7	Reinfected	SDX	9	54.7	34.2-75.2	44.4	29.4-104.0
7	Not reinfected	SDX	20	50.5	46.2-54.9	52.7	33.3-64.7
7	Reinfected	PYM	6	158.9	112.9-204.9	150.2	111.7-227.5
7	Not reinfected	PYM	18	160.9	132.3-189.5	158.8	78.0- 282.1

SP=Sulfadoxine Pyrimethamine, AS= Artesunate, PYR=Pyrimethamine, SDX=Sulfadoxine,

Table 2- A summary of the mean and median parasitaemia (µg/ml) on day 0 before receiving the drug by reinfection outcome shown separately for SP and SPAS treatment arms.

Day	Outcome	Drug arm	N	Mean parasitaemia µg/ml	Confidence Interval	Median parasitaemia µg/ml	2.5th -97.5th percentiles
0	Reinfected	SP+Placebo	35	95,897.3	75,879.4 - 115,915.3	93873.0	2,742.5 - 189,450.7
0	Not reinfected	SP+Placebo	79	89,927.9	76,626.8 - 103,229.1	90196.0	2,270.0 - 191,750.3
0	Reinfected	SPAS	26	77,410.8	49,594.2 - 105,227.4	62583.5	2,061.5 - 196,217.5
0	Not reinfected	SPAS	88	71,178.8	58,929.5 - 83,428.1	74259.0	2,064.2 189,739.5

SP=Sulfadoxine Pyrimethamine, AS= Artesunate

Table 3 - Cox proportional hazards regression model outputs of the crude and adjusted effects of drug concentrations and initial parasite density on to reinfection. Crude hazard ratios are obtained from a univariable model, and adjusted Hazard Ratios are obtained from a model accounting for all three variables : Day 0 parasitaemia, Day 0 sulfadoxine concentration, and Day 0 pyrimethamine concentration. P-values are two-sided, calculated using the Wald test. No adjustments for multiple comparisons were applied.

**Drug arm**	**Factor**	**Crude Hazard Ratio (95%Confidence Interval)**	**p-value**	**Adjusted Hazard Ratio (95%Confidence Interval)**	**p-value**
SP+Placebo	Day 0 Parasitaemia	1.01(0.96-1.07)	0.64	0.99(0.89-1.10)	0.82
	Day 0 SDX concentration	0.99(0.87-1.11)	0.82	1.11(0.91-1.37)	0.28
	Day 0 PYM concentration	0.98(0.94-1.03)	0.50	0.96(0.89-1.04)	0.32
SPAS	Day 0 Parasitaemia	1.02(0.96-1.09)	0.49	1.04(0.92-1.18)	0.51
	Day 0 SDX concentration	1.03 (0.94-1.13)	0.48	1.09(0.97-1.23)	0.15
	Day 0 PYM concentration	0.99(0.96-1.02)	0.47	0.97(0.92-1.01)	0.14

Hazard ratios shown for parasitaemia are per 10,000 parasite increase;
Hazard ratios shown for drug concentrations per 10 µg/ml increase;
SP=Sulfadoxine Pyrimethamine, AS= Artesunate, PYR=Pyrimethamine, SDX=Sulfadoxine,
Day 0 parasitaemia is measured prior to receiving the drug, whereas day 0 drug concentrations are measured on the same day after receiving the drug.

Supplementary Figure 3: Protective efficacy and duration of protection for SPAQ, CQ and SP-CQ

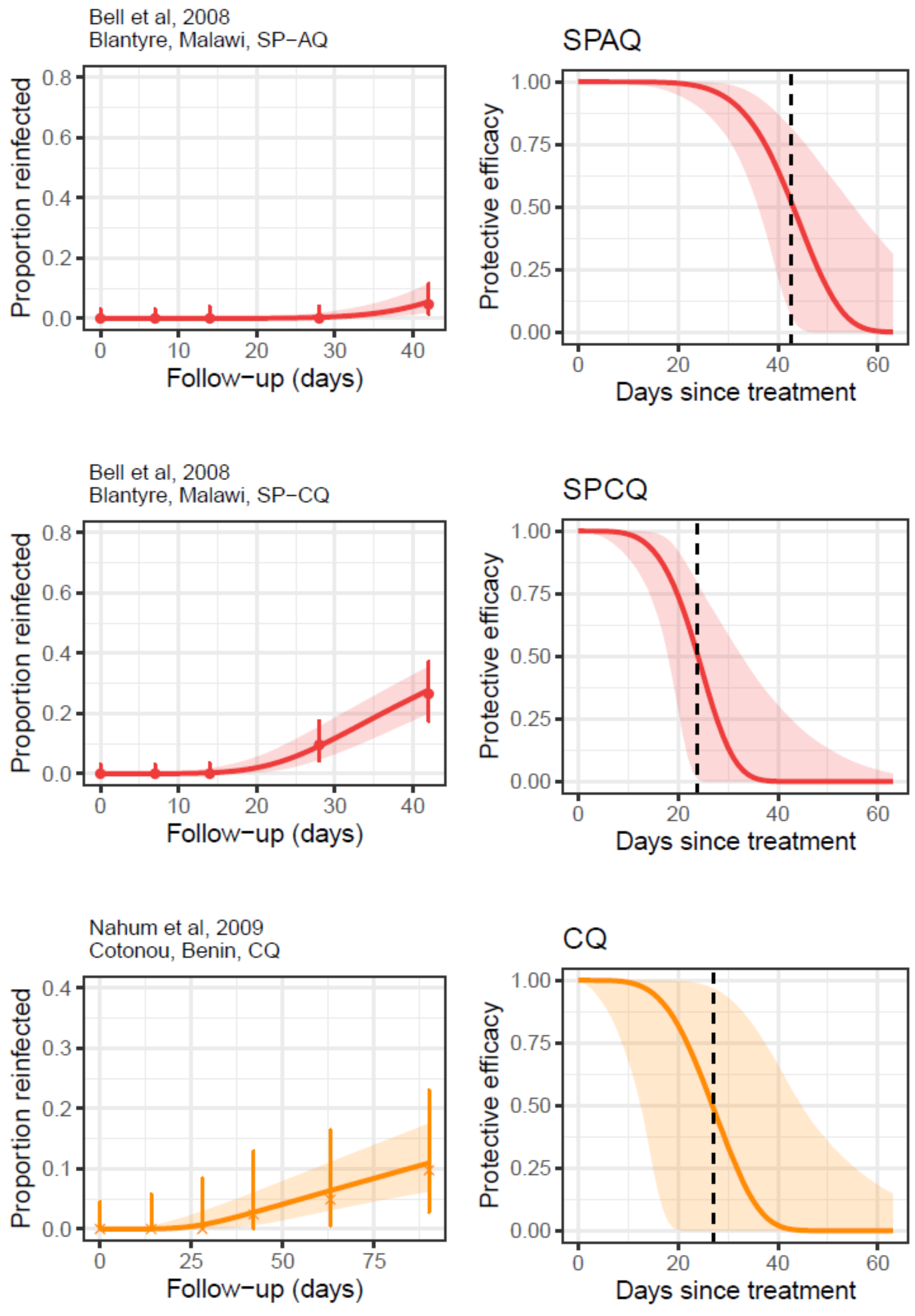

The panels on the left show the model fits to the following drug groups: SP-AQ, SP-CQ and CQ. The SP-AQ and SP-CQ drug arms were both in a setting consisting of almost entirely quintuple genotypes (shown in red). The chloroquine arm was set in an area of mostly dhps GKA (437G-K540-A581)genotypes (in orange). The panels on the right show the probability of protection (protective efficacy) since treatment shown for each drug. The vertical line denotes the mean duration of protection provided by the drug against each genotype: SP + amodiaquine (SPAQ)= 42.5 days, SP + chloroquine (SPCQ)= 23.8 days, chloroquine (CQ)= 27.0 days. Refer to Table 1 for sample size in each trial arm.

Supplementary Note 3: Web interface

An interactive tool for estimating the median duration of protection and the 30-day protective efficacy following an SP dose can be accessed at: https://andriamousa.shinyapps.io/SP_PE_prediction_tool/

Mean duration of protection against a specific strain can be easily derived using the estimated Weibull scale and shape parameters (for more details see Supplementary Note 1). In the presence of more than one strain, the mean duration of protection can not be easily derived. The prediction tool outputs the median duration of protection against any parasite given the frequencies of each strain, defined as the time since SP dose when protective efficacy drops to 50%.

In this tool, we present 30-day protective efficacy against first infection, and 30 day protective efficacy against any infection accounting for potential multiple episodes in the same individual. Throughout the manuscript, we report 30-day protective efficacy against first infection, which was estimated as the percentage of new infections prevented by the drug compared to no chemoprevention:

$$1-\frac{I_{day30\ (chemoprevention)}}{I_{day30\ (no\ chemoprevention)}},$$

where I_{day30} is the cumulative proportion infected by day 30 in each arm. More details on the deterministic model used for obtaining these proportions can be found elsewhere.[1]

In figure 6 and in the web-tool, we also report 30-day efficacy against *any* infection, accounting for potential reinfections, estimated as:

$$\sum_{X\in\begin{Bmatrix}AKA\\GKA\\GEA\\GEG\\other\end{Bmatrix}} F_X\left(\frac{1}{30}\sum_{t=1}^{30} e^{-\left(\frac{t}{\lambda_X}\right)^{w_X}}\right)$$

Where F_X is the frequency each genotype X for all *dhps* combinations at the 437, 540, 581 loci. w_X and λ_X represent the shape and scale parameters from the Weibull curve of protective efficacy over time against a particular genotype X.

This tool uses the estimated SPAS parameters from the combined fit, which are specific to each genotype. For the *dhps* GEG genotype (*dhps* 437G/540E/581G), we used the estimated parameters for the SP arm (as no GEG mutants were found in the analysed trials of SPAS). The frequency of *dhps* genotypes other than those estimated are denoted at the bottom of the inputs section as "other *dhps* combinations" (*dhps* AKG, AEG, AEA, GKG) . For this group, the tool assumes a scale parameter of 23 and a shape parameter of 4.5 (median duration of protection of 21.2 days). However, these parasite variants are likely to be rare in most settings.

Supplementary Figure 4: Frequency estimates of dhps haplotypes across sub-Saharan Africa

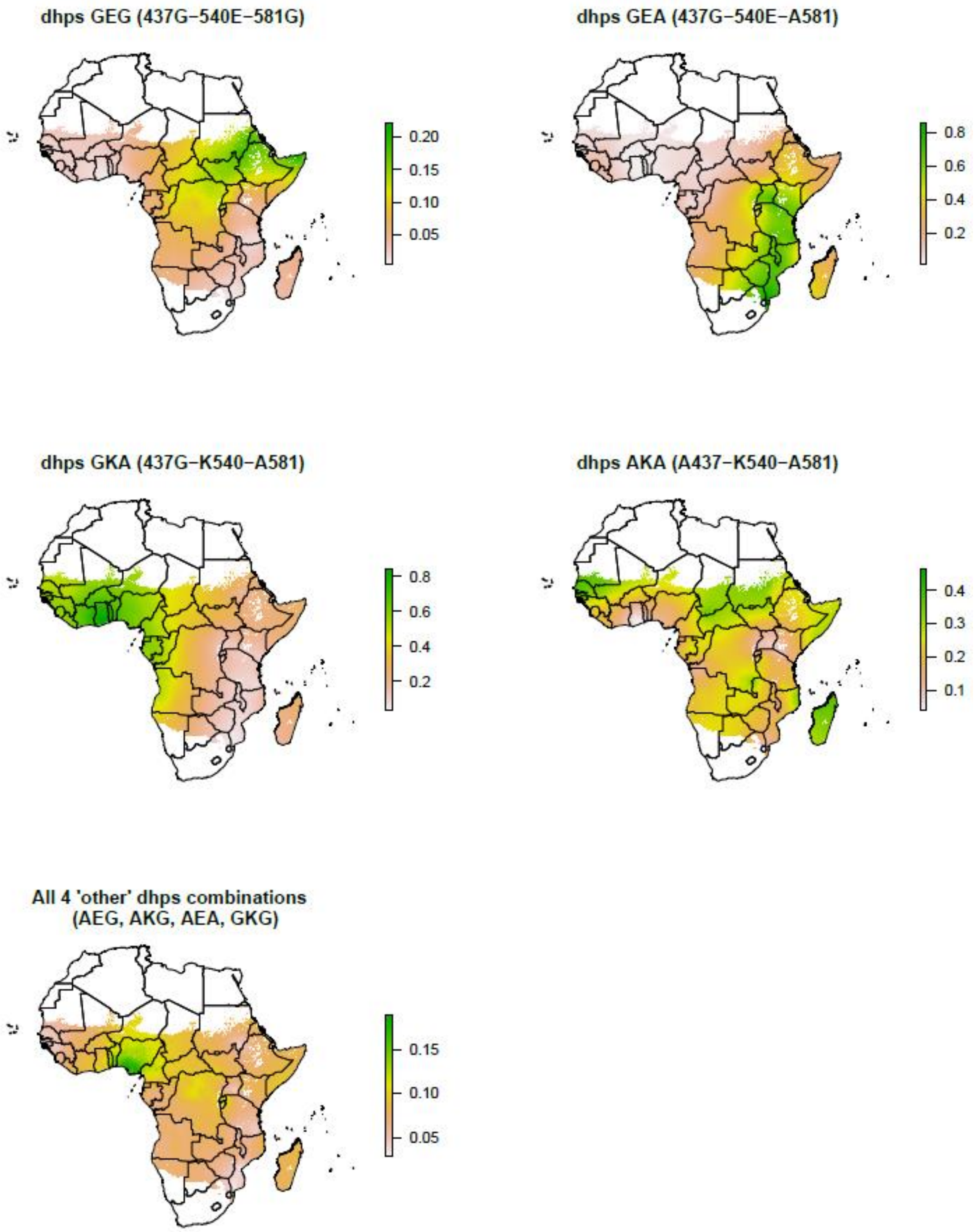

The maps shown here are model predictions of genotype frequencies for all combinations of *dhps* 437, 540 and 581 across sub-Saharan Africa for 2020, based on an already published spatiotemporal Bayesian model by Foo and Flegg.[14]

Supplementary Figure 5: Impact of a single SP dose across sub-Saharan Africa

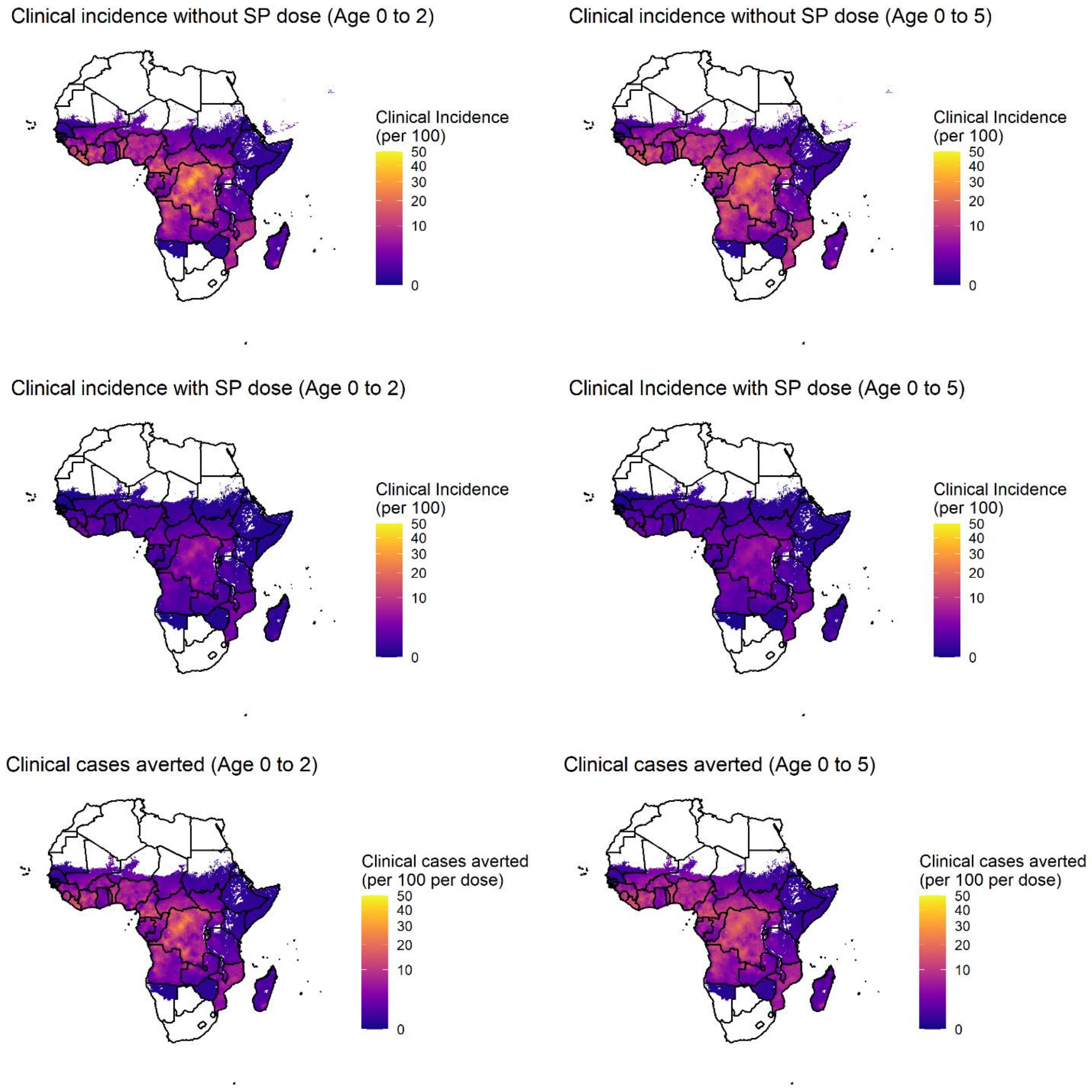

The top row shows the predicted clinical malaria incidence (per 100 children per dose) for ages 0 to 2 and ages 0 to 5, using Malaria Atlas Project prevalence estimates in 2 to 10 year olds, and the relationship between prevalence and incidence in those two age groups obtained from a published malaria transmission model developed in Imperial College, London[10,15]. We assumed a moderate antimalarial treatment coverage of 10-30% based on the Demographic Health Surveys, and smoothed the relationship between incidence and prevalence using LOESS regression. The second row of maps shows the equivalent clinical malaria incidence in these two age groups, following a single dose of SP. The number of clinical cases averted (per 100 children per dose) for the two age groups is shown in the bottom row.

Supplementary Table 6: Model diagnostics

Parameter	Dhps genotype (for genotype-specific parameter) A437G-K540E-A581G	Drug (for drug-specific parameters)	Site (for site-specific parameters)	R-hat	Effective Sample Size (ESS)	ESS bulk	ESS tail
Malaria Incidence	−	−	Blantyre, Malawi, 2003-2005 (Bell et al., 2008)	1.001	5944.482	20297	19688
	−	−	Tanga Region, Northern Tanzania, 2006 (Gesase et al., 2009)	1.001	15541.216	19234	19380
	−	−	Cotonou, Benin, 2003-2005 (Nahum et al., 2007; Nahum et al., 2009)	1.000	17431.398	19862	20311
	−	−	Magude, Mozambique, 2004-2005 (Allen et al., 2009)	1.000	19867.4	19999	19836
	−	−	Boane, Mozambique, 2004-2005 (Allen et al., 2009)	1.000	18877.458	19717	19355
	−	−	Namaacha, Mozambique, 2003 (Allen et al., 2009)	1.000	21109.625	20555	19888
	−	−	Catuane, Mozambique, 2003 (Allen et al., 2009)	1.000	18644.314	19120	19397
	−	−	Namaacha, Mozambique, 2002 (RHXNJ) (Barnes et al., 2006)	1.000	13410.102	19053	19381
	−	−	Bela Vista Mozambique, 2002 (RZENT) (Barnes et al., 2006)	1.000	22440.979	20191	19507
	−	−	Bela Vista, Mozambique, 2003 (NMMSB) (Barnes et al., 2006)	1.000	22583.542	19855	19047
	−	−	Mpumalanga, South Africa, 2002 (PEADD) (Barnes et al., 2008 ; Mabuza et al., 2005)	1.000	22332.454	19366	19384
	−	−	Ndumu, KwaZulu-Natal, South Africa, 2000 (Bredenkamp et al., 2001)	1.000	22674.584	20019	19996

Supplementary Table 6: Model diagnostics (continued)

Parameter	Dhps genotype (for genotype-specific parameter) A437G-K540E-A581G	Drug (for drug-specific parameters)	Site (for site-specific parameters)	R-hat	Effective Sample Size (ESS)	ESS bulk	ESS tail
Frequency	GEA	−	Magude, Mozambique, 2004-2005	1.000	14960.719	19536	19338
	GEA	−	Boane, Mozambique, 2004-2005	1.000	19877.375	19543	19129
	GEA	−	Namaacha, Mozambique, 2003	1.000	20655.268	19690	19269
	GEA	−	Catuane, Mozambique, 2003	1.000	25220.748	20111	20041
	GEA	−	Namaacha, Mozambique, 2002 (RHXNJ)	1.000	17685.834	19752	19232
	GEA	−	Bela Vista Mozambique, 2002 (RZENT)	1.000	20799.341	19825	18695
	GEA	−	Bela Vista, Mozambique, 2003 (NMMSB)	1.000	23003.141	20043	20008
	GEA	−	Mpumalanga, South Africa, 2002 (PEADD)	1.000	23366.808	19778	19597
	Sextuple	−	Tanga Region, Northern Tanzania, 2006	1.000	18998.43	20473	19277
Probability of determining genotype for new infection	−	−	Tanga Region, Northern Tanzania, 2006	1.000	21939.326	20132	19677
Scale parameter corresponding to the Weibull distribution of protective efficacy over time	AKA	SP	−	1.000	13572.33	20072	19777
	GKA		−	1.000	20381.31	20070	19554
	GEA		−	1.001	9055.82	19466	18636
	Sextuple		−	1.000	9586.417	19867	19655
Shape parameter corresponding to the Weibull distribution of protective efficacy over time	AKA		−	1.000	18131.71	19451	18038
	GKA		−	1.000	23145.667	19727	20037
	GEA		−	1.001	6291.623	18528	19389
	Sextuple		−	1.000	14264.942	19953	19970
Scale parameter	AKA	SPAS	−	1.000	14945.99	18890	18522
	GKA		−	1.000	20298.07	19573	19994
	GEA		−	1.001	5121.386	19973	19103
Shape parameter	AKA		−	1.000	19050.119	20113	19496
	GKA		−	1.000	22556.417	19612	19680
	GEA		−	1.001	7646.452	19836	18968
Scale parameter	GKA	CQ	−	1.000	22354.24	19695	19725
Shape parameter	GKA		−	1.000	22886.123	19879	19841
Scale parameter	GEA	SPCQ	−	1.000	8838.647	19945	19113
Shape parameter	GEA		−	1.000	15058.59	20134	19492
Scale parameter	GEA	SPAQ	−	1.000	12786.717	19025	18964
Shape parameter	GEA		−	1.000	17573.32	18602	18988

Supplementary Table 7 Sensitivity analysis accounting for heterogeneity in risk of transmission.

Model-estimated duration of protection by each drug against each dhps genotype for the main analysis and sensitivity analysis accounting for heterogeneity in the risk of transmission. Estimates are based on individual participant data with a total sample size of 1,639 across 12 trial sites.

Drug group	*dhps* genotype (A437**G**/K540**E**/A581**G**)	Mean duration of protection in days (Median and 95% Credible Interval)	
		**Main analysis**	**Sensitivity analysis**
**Sulfadoxine-Pyrimethamine (SP)**			
	AKA (sulfadoxine-susceptible)	55.7 (46.9 - 71.6)	54.9 (45.1 – 74.8)
	**G**KA	33.9 (16.8 - 56.8)	34.0 (14.5 – 62.0)
	**GE**A	10.7 (8.9 - 21.9)	10.3 (6.6 – 15.6)
	**GEG**	11.7 (8.0 - 21.9)	10.5 (6.8 - 18.8)
**Sulfadoxine-Pyrimethamine (SP) + Artesunate (SPAS)**			
	AKA (sulfadoxine-susceptible)	56.0 (46.8 - 72.1)	55.5 (45.1 – 75.7)
	**G**KA	30.3 (17.1 - 45.1)	30.4 (14.8 – 48.7)
	**GE**A	16.5 (11.2 - 37.4)	15.0 (10.7 - 37.0)
**Sulfadoxine-Pyrimethamine (SP) + Amodiaquine (SPAQ)** †			
	**GE**A	42.5 (36.7 - 52.4)	43.0 (35.7 – 56.0)
**Sulfadoxine-Pyrimethamine + Chloroquine (SPCQ)** †			
	**GE**A	23.8 (18.8 - 31.4)	23.7 (17.5 – 32.1)
**Chloroquine (CQ)**			
	**G**KA	27.1 (14.8 - 41.9)	27.1 (13.0 – 45.4)

SP: sulfadoxine pyrimethamine, SPAS: SP + artesunate, SPAQ: SP + amodiaquine, SPCQ: SP + chloroquine, CQ: chloroquine

†day 0 prevalence of *Pfcrt 76T, Pfmdr1 86Y,* and *Pfmdr1 1246Y* mutations were low (0%, ~10%, and 3%, respectively) in the study conducted in Malawi

Supplementary Figure 6: Estimated incidence of malaria infection

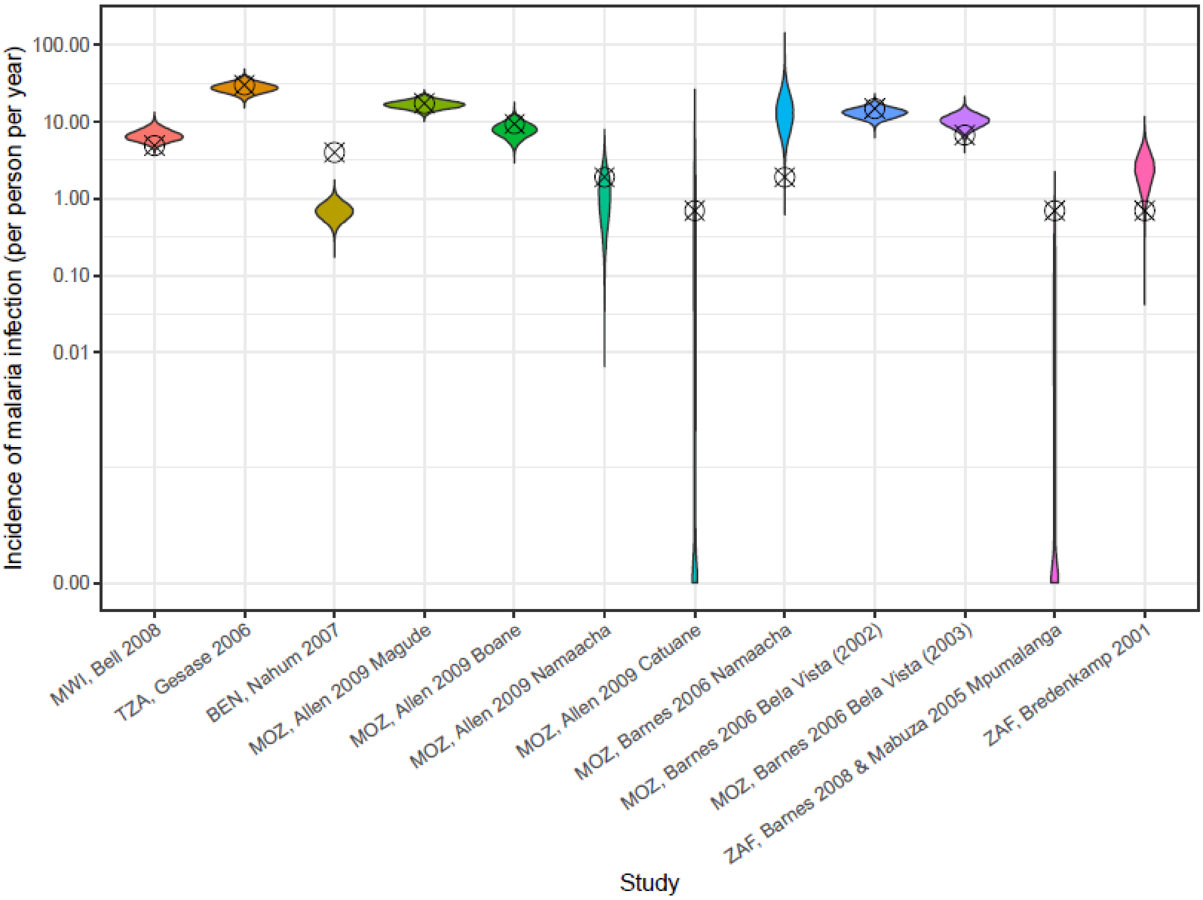

The violin plots show the posterior distribution of incidence of malaria infection (per person per year) estimated for each trial site. The markers denote the prior values used in the model (see details in the table above on “Prior distributions and posterior estimates”.

References

1. Mousa A, Cuomo-Dannenburg G, Thompson HA, et al. Measuring protective efficacy and quantifying the impact of drug resistance: A novel malaria chemoprevention trial design and methodology. *PLOS Medicine* 2024; **21**(5): e1004376.
2. Bell DJ, Nyirongo SK, Mukaka M, et al. Sulfadoxine-pyrimethamine-based combinations for malaria: a randomised blinded trial to compare efficacy, safety and selection of resistance in Malawi. *PLoS One* 2008; **3**(2): e1578.
3. Roper C, Pearce R, Bredenkamp B, et al. Antifolate antimalarial resistance in southeast Africa: a population-based analysis. *The Lancet* 2003; **361**(9364): 1174-81.
4. Gesase S, Gosling RD, Hashim R, et al. High resistance of Plasmodium falciparum to sulphadoxine/pyrimethamine in northern Tanzania and the emergence of dhps resistance mutation at Codon 581. *PLoS One* 2009; **4**(2): e4569.
5. Allen EN, Little F, Camba T, et al. Efficacy of sulphadoxine-pyrimethamine with or without artesunate for the treatment of uncomplicated Plasmodium falciparum malaria in southern Mozambique: a randomized controlled trial. *Malar J* 2009; **8**: 141.
6. Barnes KI, Little F, Smith PJ, Evans A, Watkins WM, White NJ. Sulfadoxine-pyrimethamine pharmacokinetics in malaria: Pediatric dosing implications. *Clinical Pharmacology & Therapeutics* 2006; **80**(6): 582-96.
7. Barnes KI, Little F, Mabuza A, et al. Increased Gametocytemia after Treatment: An Early Parasitological Indicator of Emerging Sulfadoxine-Pyrimethamine Resistance in Falciparum Malaria. *The Journal of Infectious Diseases* 2008; **197**(11): 1605-13.
8. Mabuza A, Govere J, La Grange K, et al. Therapeutic efficacy of sulfadoxine-pyrimethamine for Plasmodium falciparum malaria. *S Afr Med J* 2005; **95**(5): 346-9.
9. Griffin JT, Cairns M, Ghani AC, et al. Protective Efficacy of Intermittent Preventive Treatment of Malaria in Infants (IPTi) Using Sulfadoxine-Pyrimethamine and Parasite Resistance. *PLOS ONE* 2010; **5**(9): e12618.
10. Griffin JT, Ferguson NM, Ghani AC. Estimates of the changing age-burden of Plasmodium falciparum malaria disease in sub-Saharan Africa. *Nat Commun* 2014; **5**: 3136.
11. Weiss DJ, Lucas TCD, Nguyen M, et al. Mapping the global prevalence, incidence, and mortality of Plasmodium falciparum, 2000-17: a spatial and temporal modelling study. *Lancet* 2019; **394**(10195): 322-31.
12. Pfeffer DA, Lucas TCD, May D, et al. malariaAtlas: an R interface to global malariometric data hosted by the Malaria Atlas Project. *Malaria Journal* 2018; **17**(1): 352.
13. Bell DJ, Wootton D, Mukaka M, et al. Measurement of adherence, drug concentrations and the effectiveness of artemether-lumefantrine, chlorproguanil-dapsone or sulphadoxine-pyrimethamine in the treatment of uncomplicated malaria in Malawi. *Malaria Journal* 2009; **8**(1): 204.
14. Foo YS, Flegg JA. A spatio-temporal model of multi-marker antimalarial resistance. *J R Soc Interface* 2024; **21**(210): 20230570.
15. Griffin JT, Hollingsworth TD, Okell LC, et al. Reducing Plasmodium falciparum Malaria Transmission in Africa: A Model-Based Evaluation of Intervention Strategies. *PLOS Medicine* 2010; **7**(8): e1000324.
