# Supplementary material for: Impact of dhps mutations on sulfadoxine-pyrimethamine protective efficacy and implications for malaria chemoprevention
Source: Nat Commun. 2025 May 8;16:4268. doi: 10.1038/s41467-025-58326-z (PMC12062426; doi:10.1038/s41467-025-58326-z)
Supplement: Supplementary file 3 — Reporting Summary [file 41467_2025_58326_MOESM3_ESM.pdf]

nature portfolio

Corresponding author(s): Andria Mousa

Last updated by author(s): Jan 16, 2025

Reporting Summary

Nature Portfolio wishes to improve the reproducibility of the work that we publish. This form provides structure for consistency and transparency in reporting. For further information on Nature Portfolio policies, see our Editorial Policies and the Editorial Policy Checklist.

Statistics

For all statistical analyses, confirm that the following items are present in the figure legend, table legend, main text, or Methods section.

n/a	Confirmed	
☐	☒	The exact sample size (*n*) for each experimental group/condition, given as a discrete number and unit of measurement
☐	☒	A statement on whether measurements were taken from distinct samples or whether the same sample was measured repeatedly
☐	☒	The statistical test(s) used AND whether they are one- or two-sided<br>*Only common tests should be described solely by name; describe more complex techniques in the Methods section.*
☐	☒	A description of all covariates tested
☐	☒	A description of any assumptions or corrections, such as tests of normality and adjustment for multiple comparisons
☐	☒	A full description of the statistical parameters including central tendency (e.g. means) or other basic estimates (e.g. regression coefficient) AND variation (e.g. standard deviation) or associated estimates of uncertainty (e.g. confidence intervals)
☐	☒	For null hypothesis testing, the test statistic (e.g. *F*, *t*, *r*) with confidence intervals, effect sizes, degrees of freedom and *P* value noted<br>*Give P values as exact values whenever suitable.*
☐	☒	For Bayesian analysis, information on the choice of priors and Markov chain Monte Carlo settings
☐	☒	For hierarchical and complex designs, identification of the appropriate level for tests and full reporting of outcomes
☒	☐	Estimates of effect sizes (e.g. Cohen's *d*, Pearson's *r*), indicating how they were calculated

Our web collection on statistics for biologists contains articles on many of the points above.

Software and code

Policy information about availability of computer code

Data collection	All data used in this manuscript are secondary data from studies already in the public domain. Data cleaning and management was done using R version 4.0.3 and can be accessed via the GitHub directory : https://github.com/AndriaMousa/SP-resistance-protective-efficacy-code
Data analysis	All analyses used in this manuscript were done using R version 4.0.3 and can be accessed via the GitHub directory : https://github.com/AndriaMousa/SP-resistance-protective-efficacy-code

For manuscripts utilizing custom algorithms or software that are central to the research but not yet described in published literature, software must be made available to editors and reviewers. We strongly encourage code deposition in a community repository (e.g. GitHub). See the Nature Portfolio guidelines for submitting code & software for further information.

Data

Policy information about availability of data

All manuscripts must include a data availability statement. This statement should provide the following information, where applicable:

- Accession codes, unique identifiers, or web links for publicly available datasets
- A description of any restrictions on data availability
- For clinical datasets or third party data, please ensure that the statement adheres to our policy

The data that support the findings of this study are available with no restrictions in: https://github.com/AndriaMousa/SP-resistance-protective-efficacy-code

Research involving human participants, their data, or biological material

Policy information about studies with human participants or human data. See also policy information about sex, gender (identity/presentation), and sexual orientation and race, ethnicity and racism.

Reporting on sex and gender	This is a secondary analysis of clinical trials and data on gender/sex were not available for most of the studies included. We anticipate that the effect of gender or sex or the risk of malaria infection will be minimal. The ages of participants are summarized in the manuscript and in Table 1.
Reporting on race, ethnicity, or other socially relevant groupings	No information on social group variables was available from primary studies included.
Population characteristics	All relevant covariates, which were available from the original studies, are summarised and/or included in the main analyses or sensitivity analyses. These include age, drug administered, clinical malaria outcome, drug levels and genotype of parasite infections. These are all detailed in the manuscript
Recruitment	Recruitment of participants is detailed in the original studies and summarized in this manuscript and supplementary materials.
Ethics oversight	All original trials have been published and approved by both institutional and local ethics review committees. This secondary analysis has been approved by the London School of Hygiene and Tropical Medicine ethics Committee (reference 29340)

Note that full information on the approval of the study protocol must also be provided in the manuscript.

Field-specific reporting

Please select the one below that is the best fit for your research. If you are not sure, read the appropriate sections before making your selection.

[x] Life sciences   [ ] Behavioural & social sciences   [ ] Ecological, evolutionary & environmental sciences

For a reference copy of the document with all sections, see nature.com/documents/nr-reporting-summary-flat.pdf

Life sciences study design

All studies must disclose on these points even when the disclosure is negative.

Sample size	No formal sample size calculations were performed, as this study analyses secondary data. Study inclusion was not based on sample size criteria. The smallest included study had 23 participants, which was deemed sufficient. All data were analysed using a single model, where smaller sample sizes contribute proportionally less weight.
Data exclusions	All available data were analysed with no exclusions.
Replication	All analysis and findings are reproducible using the code and data provided
Randomization	Not applicable here as this is a secondary data analysis of published trials.
Blinding	Not applicable here as this is a secondary data analysis of published trials.

Reporting for specific materials, systems and methods

We require information from authors about some types of materials, experimental systems and methods used in many studies. Here, indicate whether each material, system or method listed is relevant to your study. If you are not sure if a list item applies to your research, read the appropriate section before selecting a response.

Materials & experimental systems

n/a	Involved in the study	
[x]	[ ]	Antibodies
[x]	[ ]	Eukaryotic cell lines
[x]	[ ]	Palaeontology and archaeology
[x]	[ ]	Animals and other organisms
[x]	[ ]	Clinical data
[x]	[ ]	Dual use research of concern
[x]	[ ]	Plants

Methods

n/a	Involved in the study	
[x]	[ ]	ChIP-seq
[x]	[ ]	Flow cytometry
[x]	[ ]	MRI-based neuroimaging

Plants

Seed stocks	N/A
Novel plant genotypes	N/A
Authentication	N/A
